# Real-time and video-recorded pain assessment in beef cattle: clinical application and reliability in young, adult bulls undergoing surgical castration

**DOI:** 10.1038/s41598-024-65890-9

**Published:** 2024-07-02

**Authors:** Rubia Mitalli Tomacheuski, Alice Rodrigues de Oliveira, Pedro Henrique Esteves Trindade, Magdiel Lopez-Soriano, Victoria Rocha Merenda, Stelio P. Loureiro Luna, Monique D. Pairis-Garcia

**Affiliations:** 1 Translational Research in Pain, Department of Clinical Sciences, College of Veterinary Medicine, North Carolina State University (NCSU), Raleigh, NC USA; 2 Department of Anatomy, Pathology and Veterinary Clinics, School of Veterinary Medicine and Animal Science, Federal University of Bahia (UFBA), Salvador, Bahia, Brazil; 3 Department of Population Health and Pathobiology, College of Veterinary Medicine, North Carolina State University (NCSU), Raleigh, NC USA; 4 Department of Veterinary Surgery and Animal Reproduction, School of Veterinary Medicine and Animal Science, São Paulo State University (Unesp), Botucatu, São Paulo Brazil

**Keywords:** Clinical trials, Animal behaviour

## Abstract

Bovine pain assessment relies on validated behavioral scales related to normal and pain-related behaviors. This study investigated the reliability and applicability of real-time and video-recorded pain assessment, and their agreement, in young, adult bulls undergoing surgical castration. Ten Nelore and nine Angus bulls underwent general anesthesia and surgical castration. Three-minute real-time observations and simultaneous videos were recorded at − 48 h (M0), before sedation, under fasting (M1), after surgery, 3 h after sternal recumbency (M2), after rescue analgesia (M3) and at 24 h (M4). Animals received morphine (after M2), dipyrone (after M3), and flunixin meglumine after surgical castration (M4). Two trained evaluators assessed real-time (n = 95) and video-recorded time-points (n = 95) using the Unesp-Botucatu Cattle Pain Scale (UCAPS). Both assessment methods inferred ‘very good’ reliability (≥ 0.81) with minimal bias, however, video-recorded assessment (4.33 ± 2.84) demonstrated slightly higher scores compared to real-time (3.08 ± 2.84). The results from this study suggest that UCAPS can be used in real-time or video-recorded to assess pain and guide analgesic therapy in cattle.

## Introduction

Society is increasingly demanding better husbandry practices and welfare for food-producing animals^1^. In this regard, managing pain is a core principle to guarantee basic animal welfare^2^. However, pain assessment, recognition and treatment are a significant challenge in the livestock industries^3^ given limited educational resources guiding veterinarians on appropriate pain management^3,4^ and inaccessibility of medications safe for food-producing animals, such as cattle^5^.

The issue of pain detection in animals, particularly in beef cattle, warrants careful consideration. In the context of beef production, various painful procedures such as dehorning, castration, and branding are commonly performed, contributing to welfare concerns^5,6^. In Brazil and elsewhere, a significant percentage of beef cattle undergo these procedures without adequate pain management protocols in place^7,8^. This lack of management stems from a range of factors including economic considerations, logistical challenges, and a historical lack of emphasis on animal welfare in agricultural practices^3,5,9^. Despite increasing awareness and advocacy for improved animal welfare standards, the current state of pain management for beef cattle remains insufficient^3,5,7^. Addressing this problem requires a comprehensive understanding of pain detection methods, implementation of effective pain management strategies, and a shift towards prioritizing animal welfare alongside production objectives in the beef industry^1,3,10–12^.

Pain assessment in animals is typically performed by evaluating changes to behaviour and/or facial expression^10,13^. For either approach, pain assessment instruments must be validated for use^10,14^, robust and flexible enough to be applied across different study designs and must be species-specific utilizing behaviors typical to the repertoire of the species^13,15–22^. Specific to cattle, several pain scales can be found in the literature^16,23–26^; however, these scales differ regarding study design use and validation process. Additional methods to assess pain include quantitative sensory testing to assess sensory profiles and kinetics or kinematics to evaluate activity levels and lameness^27,28^. However, these methods necessitate specialized equipment and training and may not readily evaluate the emotional dimensions of pain. Surrogate measures such as animal production outcomes, physiological parameters, and biomarkers are also used, yet they may not be specifically indicative of pain^29,30^.

The Unesp-Botucatu Cattle Pain Scale (UCAPS) developed for beef cattle^26^ is considered the most robust cattle-specific tool due to its high strength of evidence^10^. The UCAPS was developed and validated using pre-recorded videos in which observers assessed pain post-hoc as used in several studies in different species such as cattle, sheep, pigs, rabbits, and cats^18,19,21,26,31,32^. However, relying on video-recordings is a limiting factor for assessing pain in cattle given veterinarians and farmers have limited access and financial capabilities to install video equipment. In addition, assessing pain via video delays intervention opportunities to provide analgesic intervention to cattle in pain. Even though, several bovine pain scales used real-time assessment^23,25,33,34^ their strength of evidence varies between very low and moderate^10^. Hence, exploring opportunities to implement and validate the UCAPS for real-time assessment is needed. Therefore, this study aimed to investigate the reliability and the agreement of real-time and video-recorded pain assessment in beef cattle using the UCAPS on young, adult bulls undergoing surgical castration. Similar studies have been conducted in pigs, rats, and mice^35–37^. Our hypothesis proposes that there is no difference in reliability, and there is concordance in agreement between real-time and video-recorded assessment.

## Results

**Figure 1:**
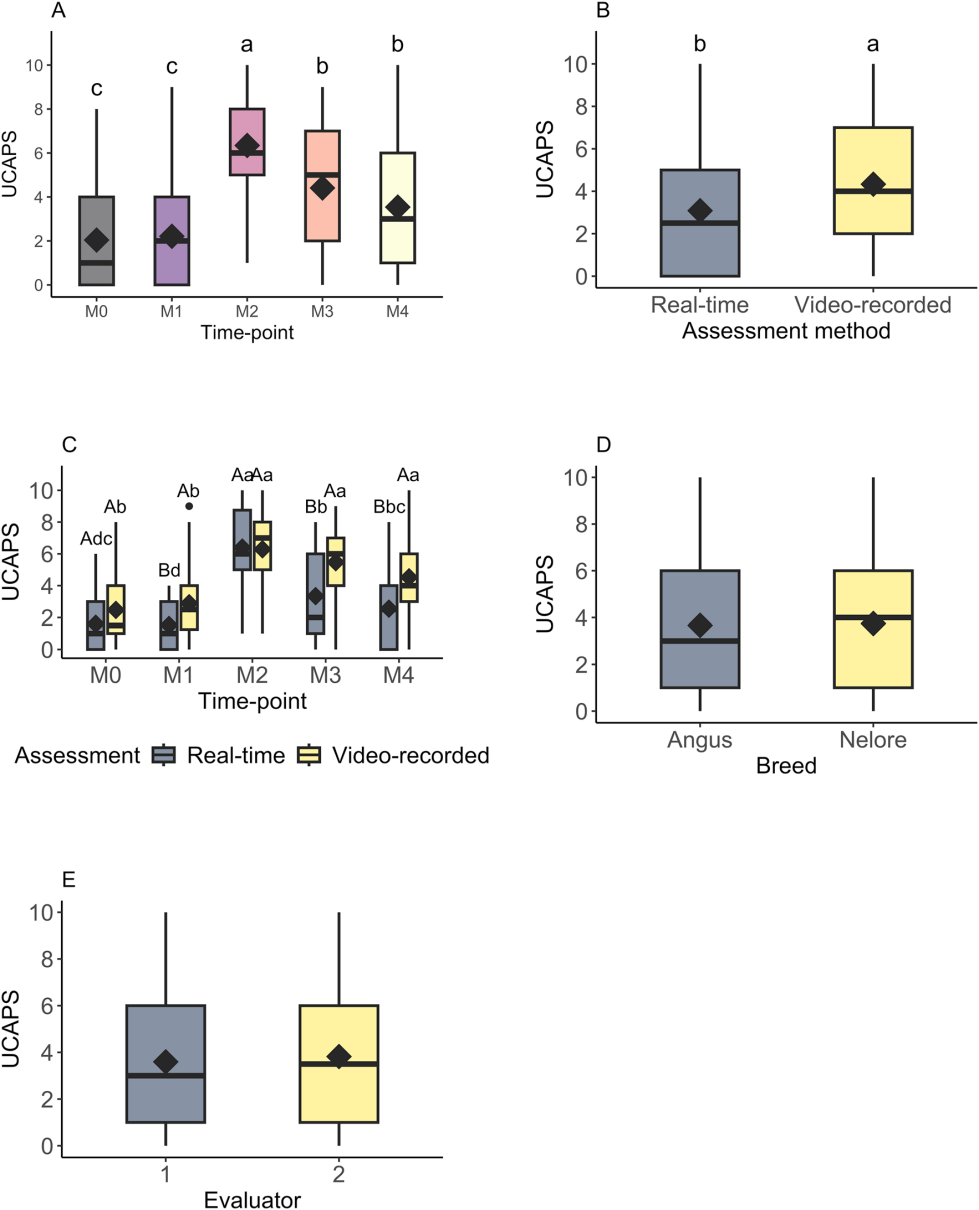
Plots of Unesp-Botucatu cattle pain scale (UCAPS) comparing time-points (**A**), assessment methods (**B**), interaction between moments, assessment methods (**C**), breed (**D**) and evaluators (**E**). The top and bottom box lines represent the interquartile range (25–75%), the line within the box represents the median, the extremes of the vertical lines represent the minimum and maximum values (mean ± 3 × standard deviation), black diamond represents the mean, black circles above or below the extremes of vertical lines represent outliers (above or below the mean ± 3 × standard deviation), different lowercase letters (a > b) indicate statistical difference (*p* < 0.05) between the time-points, while different capital letters (**A** > **B**) indicate difference (*p* < 0.05) between the assessment methods according to the multilevel zero-inflated Poisson model.

The post-hoc test for the UCAPS total scores inferred similar trends over-time (M2 > M3/M4 > M0 = M1; Fig. 1A) regardless of the assessment method. Total pain scores obtained by video-recordings were greater than the total pain scores obtained by real-time assessment (Fig. 1B) at time-points M1, M3 and M4 (Fig. 1C). All the parameters estimated by the model were described in the supplementary material (Table S1). There was no statistical difference between breed (Fig. 1D) or evaluators (Fig. 1E).

Table 1 depicts variables composing the UCAPS scale, the total scores (sum of items) for real-time and video-recorded behaviors, the statistical model implemented and the need for rescue analgesia. Video-recording assessments had higher means for locomotion, interactive behavior, and miscellaneous behavior when compared to real-time assessment (Table 1).

**Table 1:** Mean and standard-deviation of Unesp-Botucatu Cattle Pain Scale (UCAPS), the variables composing the scale, and need for rescue analgesia according to real-time and video-recorded assessments.

Variables	Real-time	Video-recorded	Model
Locomotion	**0.51 ± 0.66**	**0.71 ± 0.75**	**MGLM**
Interactive behavior	**0.39 ± 0.57**	**0.58 ± 0.69**	**MGLM**
Activity	1.06 ± 0.96	1.25 ± 0.94	MGLM
Appetite	0.70 ± 0.90	0.90 ± 0.97	MGLM
Miscellaneous behavior	**0.56 ± 0.74**	**0.90 ± 0.82**	**MGLM**
Rescue analgesia	0.29 ± 0.46	0.42 ± 0.50	MBLM
UCAPS total score	**3.08 ± 2.84**	**4.33 ± 2.84**	**MZIBN**

UCAPS total score = sum of items. MGLM: multilevel generalized linear model adjusted by Poisson distribution; MBLM: multilevel binomial logistic model; MZIBN: multilevel zero-inflated negative binomial model.

Bold is highlighting *P* < 0.05.

**Figure 2:**
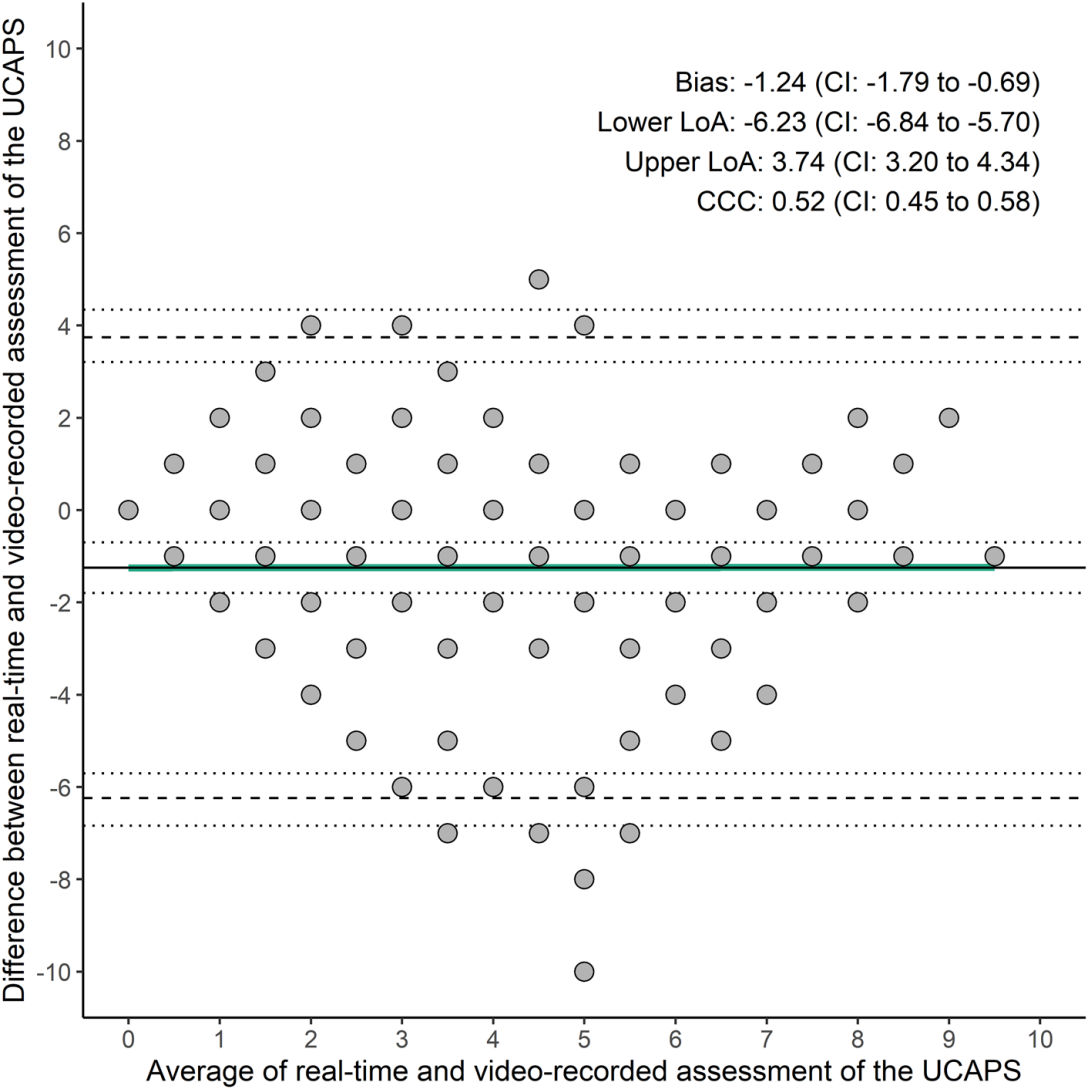
Bland–Altman plots comparing real-time and video-recorded assessment of the Unesp-Botucatu Cattle Pain Scale (UCAPS). LoA, limit of agreement; CI, 95% confidence interval; solid line represents the bias; dashed line represents the lower and upper LoA; dotted lines represent the 95% confidence interval; CCC, Lin's concordance correlation coefficient; green line is the simple linear model.

Figure 2 compares the real-time and video-recorded assessment of the Unesp-Botucatu Cattle Pain Scale (UCAPS) using Bland–Altman plots. The limit of agreement (LoA) was between − 6.24 and 3.74, with a bias of − 1.24 and a Lin's concordance correlation coefficient (CCC) of 0.52 (Fig. 2).

Table 2 depicts the percentages of LoA agreement and disagreement between real-time and video-recorded assessments using the Unesp-Botucatu Cattle Pain Scale. There was a low percentage of perfect agreement between assessment methodologies (Table 2).

**Table 2:** Percentages of LoA agreement and disagreement between real-time and video-recorded assessments.

Types	Percentage (%)
Perfect agreement	23.15
Difference within the LoA	70.52
Difference beyond the LoA	6.33

*LoA* limit of agreement based on Bland–Altman test

The slope coefficient of the mean between the two assessment methodologies (β = 0.0010) was not significant (*P* = 0.9888; Table S2), suggesting no proportional bias. The model showed homoscedasticity according to the Breusch Pagan test (X-squared = 0.2940; *P* = 0.5876).

Table 3 shows the reliability of real-time and video-recorded assessments for UCAPS and the need for rescue analgesia. The reliability between real-time *versus* video-recorded pain assessment for rescue analgesia was ‘very good’. The need for rescue analgesia was ‘good’ and ‘reasonable’ respectively for the real-time and video-recorded assessment (Table 3).

**Table 3:** Reliability of real-time and video-recorded assessment for Unesp-Botucatu cattle pain scale (UCAPS) and need for rescue analgesia.

Variable	Method	Coefficient	Estimate	CI	*P* value
UCAPS	Real-time	ICC	0.90	0.85–0.94	< 0.0001
UCAPS	Video-recorded	ICC	0.81	0.71–0.87	< 0.0001
Rescue Analgesia	Real-time	Weighted Kappa	0.70	0.53–0.84	NA
Rescue Analgesia	Video-recorded	Weighted Kappa	0.48	0.28–0.66	NA

*ICC* intraclass correlation coefficient, *CI* 95% confidence interval. The interpretation of ICC or weighted kappa was ‘very good’ 0.81–1.0; ‘good’ 0.61–0.80; ‘moderate’ 0.41–0.60; ‘reasonable’ 0.21–0.4; and ‘poor’ < 0.2^38^.

Table 4 depicts the agreement on the need for rescue analgesia between the two assessment methods using UCAPS. The reliability of UCAPS was very good for both assessment methodologies.

**Table 4:** Agreement on need for rescue analgesia between the two assessment methods for UCAPS.

		Video-recorded	
		Analgesia not indicated	Analgesia indicated
Real-time	Analgesia not indicated	99	35
	Analgesia indicated	11	45

X-squared = 45.46, df = 1, *P* value < 0.0001.

## Discussion

Pain assessment in beef cattle is challenging. The implementation of a reliable and applicable pain scoring instrument that may be used with video-recordings or real-time assessment is crucial to improve pain management in cattle. This is the first study confirming the applicability of the Unesp-Botucatu Cattle Pain Scale (UCAPS) by video-recordings and validating the UCAPS by real-time assessment in a clinical setting, in young adult bulls undergoing castration. Even though perfect agreement between the real-time and video-recorded assessment methods was low, the UCAPS demonstrated a high reliability between assessment methods with minor bias and a narrow limit of agreement. No differences were observed between breeds or evaluators.

The similarity between trends over time by UCAPS scores confirmed that the instrument was able to detect and differentiate painful (M2, M3 and M4) and non-painful (M0 and M1) states by both methods of assessment. Similar to previous studies using UCAPS^16,26,39^. In addition, significant differences were found in M1, M3, and M4 when comparing the UCAPS total score between both methods. These results were different from a recent study in piglets undergoing castration, where real-time and video-recorded behavioral assessment methodologies did not significantly affect total pain scores over time^37^. The real-time assessment presented overall lower total pain scores than the video-recorded method, and at M1, a time-point before the surgical procedure, considered non-painful. These results could be explained by the fact that the evaluators were not masked to the time-points during the real-time assessment, which may have influenced their lower scoring. Otherwise, the video-recorded method showed a higher UCAPS total score at time-points M3 and M4, and for the items’ locomotion, interactive behavior, and miscellaneous behavior. One explanation for these outcomes could be the ability to pause or rewatch the videos before scoring it, gives the evaluators the chance to observe behaviors that in real-time would not be possible, due to its quick appearance, for instance. Nonetheless, no differences between the assessment methods were found in M2 (the most painful state right after the surgical procedure) which reinforces that UCAPS can detect pain-related behaviors regardless of the assessment method used.

Similarly, to studies in cats, rats, and mice^35,36,40^, the Bland–Altman for repeated measures methods demonstrated a narrow limit of agreement and a minor bias between both methods of pain assessment, reinforcing the sufficient agreement between assessment methods. Additionally, a recent study in pigs, showed no significant impact on total pain scores over time in relation to castration whether using real-time or video-recorded method^37^. The low perfect agreement between methods probably resulted from the fact that in real-time assessment the evaluators were aware of the time-points, and for the video recorded they were masked. From a beef cow’s perspective, this study is beneficial not only to veterinarians who can apply the UCAPS in real-time, but also to researchers and laboratory animal veterinarians who aim to assess pain and intervene in experiments involving cattle undergoing castration.

The UCAPS reliability was very good for real-time and video-recorded pain assessment methods which demonstrated that the UCAPS is a reliable instrument to be implemented. Even though the need for rescue analgesia had good reliability for the real-time method, there is no literature to compare it yet. Furthermore, this study inferred moderate reliability for video-recorded pain assessment in bulls, these weighted kappa results were similar to the previous studies in cattle, and inferior to sheep and^17,19^ pigs^16,18,19^. These results could be explained by the fact that evaluators were aware of the time-points during real-time, which increased the likelihood of assigning higher scores during post-castration moments. Additionally, corroborating previous findings, no differences were found between breeds or evaluators^16^. However, future studies should test the UCAPS in a lager variety of procedures, breeds, female beef and dairy cattle, and with evaluators from different genders and background, to further investigate those outcomes.

In conclusion, real-time assessment using UCAPS may be implemented by veterinarians from a clinical perspective to improve pain diagnosis and pain management in bulls undergoing castration with similar reliability to that of video-recorded assessment. Future studies should test UCAPS for different procedures, age, sex and for dairy cattle to implement the instrument in an ample clinical setting.

### Limitations

This study has limitations and should be repeated in other animals, with different ages, sex, and breed, of beef and dairy cattle, and different procedures. Even though the study had the limitation of observers being aware of time-points and the clinical state of the animals, the pain assessment provided by the UCAPS was still consistent and reliable. Another limitation was the presence of evaluators during the video recording, although there was a brief period of acclimatization for the animals in the presence of the camera and observers to mitigate this bias, this could have generated change in the animal’s behaviour. Also, the pain scoring for short versus long habituation of animals should be assessed and compared in the future. Additional limitations included the restricted number of evaluators and the fact that all evaluators were female and veterinarians. Previous research has shown that female observers tend to assign higher pain scores during pain assessments^41^. Even though the gender of the observers might have overestimated the final pain scores, this possible effect would have been applied to both video-recorded and real-time assessments, which does not impact the interpretation of the results. Furthermore, a recent study suggested that the use of three evaluators is ideal in pain assessments^10^. Therefore, future research evaluating pain assessment tools should include a larger sample size of observers from all genders, by veterinarian technicians, veterinarians with distinct levels of experience in pain assessment, and observers of different educational backgrounds. Another limitation was the missing data on intra- and inter-rater variability for the practice scoring sessions, hence future studies should conduct those tests. Finally, given that the evaluators were not masked to time-points in real-time, the scoring could have been overestimated, future studies should test UCAPS in different conditions, such as a real-time and masked experiments.

## Methods

### Ethical statement

The study was approved by the University of São Paulo State—Unesp School of Veterinary Medicine and Animal Science Ethical Committee for the Use of Animals in Research (Approval number, 0147/2018) and performed in accordance with the Guide for the Care and Use of Agricultural Animals in Research and Teaching, COSMIN and ARRIVE guidelines and recommendations^14,42–44^. Bulls enrolled in the study were part of larger experiment^45^, which contributes to one of the 3 R’s of animal experimentation (reduce)^46^.

### Animals and surgical procedure

Ten *Bos indicus*, Nelore breed (451 kg ± 41 kg; mean ± SD) and nine *Bos taurus*, Angus breed (264 kg ± 24 kg; mean ± SD), age 19–24 months were purchased from two private farms, transported and maintained separately in two groups (Nelore and Angus) at the Experimental Farm Lageado—FMVZ/Unesp. They were housed outdoors in two separate paddocks (10 × 15 m), had ad libitum access to water (automatic auto-fill tank of 1500 L; diameter of 1.5 m), and were fed with hay and grain (feeder of 10 m long, 80 cm high, 40 cm wide). They were acclimatized to this site for one month before the start of the experiment. After this period, they were transported to the FMVZ/Unesp veterinary hospital in groups of three to four animals per week, where they were maintained under similar conditions receiving the same food and water ad libitum. The animals had a varied acclimatization time at the FMVZ/Unesp veterinary hospital, according to the order and date of the procedure for each animal. The first animal of the week had the shortest acclimatization time (2–12 h) before being separated for fasting, and the other two animals of each week had a longer acclimatization time (24–72 h). The surgical part of the experiment took place from March 18th and April 29th of 2019. After the end of the experiments, the animals were kept at the Experimental Farm Lageado—FMVZ/Unesp for two months for fattening and then sent for humane slaughter.

These bulls were selected for a study assessing testicular warming^45^ and after completion of the sampling all bulls underwent surgical castration. At the FMVZ/Unesp veterinary hospital, each animal was individually fasted for water and food for 24 and 48 h, respectively, before the procedure, and a physical examination was performed. Bulls underwent general anesthesia^16^ using xylazine (0.05 mg/kg, Xilazin®, Syntec do Brasil Ltda, Santana do Parnaíba, SP, Brazil) administered intravenously (IV) and induced with ketamine (2.5 mg/kg, Dopalen®, Ceva Saúde Animal Ltda, Paulínea, SP, Brazil) and diazepam (0.05 mg/kg, Compaz®, Cristália, São Paulo, SP, Brazil) IV. The patient was positioned in lateral recumbency on the surgical table, and anesthesia plane was maintained with isoflurane (Isoforine®, Cristália, São Paulo, SP, Brazil) in oxygen (15 L/min) using a large animal anesthetic machine (Model 2800C, Mallard Medical, Redding, CA, USA). Flunixin meglumine (1.1 mg/kg, Banamine®, MSD Saúde Animal, Cruzeiro, SP, Brazil) was administered intramuscularly (IM), and xylazine (0.05 mg/kg diluted to a volume of 20 mL with saline 0.9%) was administered epidurally at the level of the sacrococcygeal intervertebral space to alleviate peri-operative and operative pain^47^. Once cattle reached a stage 3 steady anesthetic plane^48^, the scrotal area was cleaned with water and antiseptic solution (Riodeine Dermatologico Suave Tópico®, Rioquímica, São José do Rio Preto, SP, Brazil), and a bilateral scrotal incision was made using a scalpel blade and both testicles were completely removed. All animals received morphine (administrated after surgery, 3 h after sternal recumbency; 0.1 mg/kg IM, Dimorf®, Cristália Prod. Quím. Farm. Ltda., São Paulo, Brazil), and flunixin meglumine (1.1 mg/kg, Banamine®, MSD Saúde Animal, Cruzeiro, SP, Brazil) was administrated IM at 24, 48 and 72 h post-castration. The mean duration of time from the induction of anesthesia to the end of surgery was 5 h 43 ± 32 min. After the end of the procedure, the animals took 14 ± 5 min for extubating, 17 ± 7 min to spontaneously assume sternal recumbency, and 38 ± 13 min to reach the quadrupedal position.

### Pain assessment

**Figure 3:**
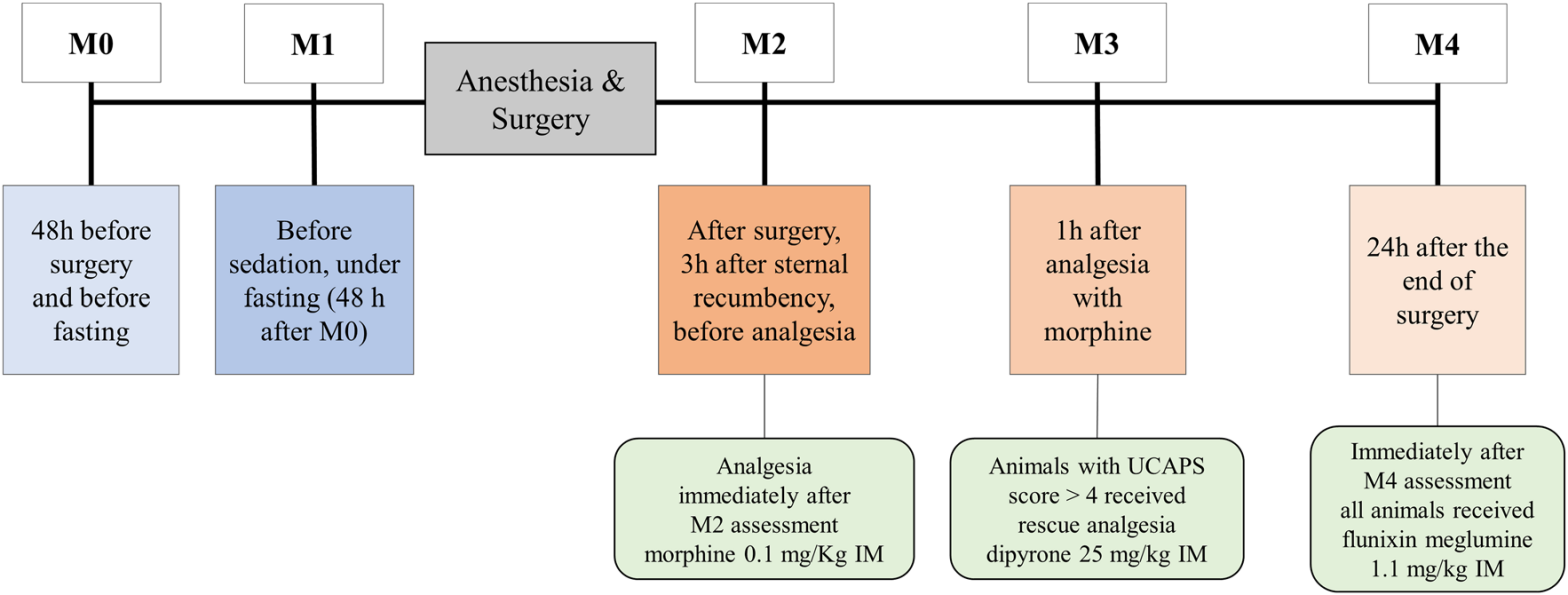
Timeline of the time-points used for the video-recorded and real-time pain assessment using the Unesp-Botucatu Cattle Pain Scale (UCAPS). Video recording was performed for 3 min at each time-point.

The pain assessment tool used in this study was the UCAPS^26^ with a maximum score of 10 (Table 5). The UCAPS total score is the sum of items. Pain behaviour was assessed continuously for three consecutive minutes at five time-points (Fig. 3).

**Table 5:** Unesp-Botucatu Cattle Pain Scale (UCAPS)^26^.

Item	Description
Locomotion	(0) Walking with no obviously abnormal gait
	(1) Walking with restriction, may be with hunched back and/or short steps
	(2) Reluctant to stand up, standing up with difficulty or not walking
Interactive behaviour	(0) Active; attention to tactile and/or visual and/or audible environmental stimuli; when near other animals, can interact with and/or accompany the group
	(1) Apathetic: may remain close to other animals but interacts little when stimulated
	(2) Apathetic: may be isolated or may not accompany the other animals; does not react to tactile, visual and/or audible environmental stimuli
Activity	(0) Moves normally
	(1) Restless, moves more than normal or lies down and stands up with frequency
	(2) Moves less frequently in the pasture or only when stimulated
Appetite	(0) Normorexia and/or rumination
	(1) Hyporexia
	(2) Anorexia
Miscellaneous behaviours	Wagging the tail abruptly and repeatedly
	Licking the affected area
	Moves and arches the back when in standing posture
	Kicking/foot stamping
	Hind limbs extended caudally when in standing posture
	Head below the line of spinal column
	Lying down in ventral recumbency with full or partial extension of one or both hind limbs
	Lying down with the head on/close to the ground
	Extends the neck and body forward when lying in ventral recumbency
	(0) All of the above-described behaviours are absent
	(1) Presence of 1 of the behaviours described above
	(2) Presence of 2 or more of the behaviours described above

### Behavioral assessment methodologies

Cattle pain was assessed using the UCAPS via two different techniques:

1. Real-time assessment
  Two veterinarians with experience in pain assessment (A.R.O. and R.M.T.) performed all real-time evaluations at each of the aforementioned time-points. Before starting the assessment, the evaluators received training and guidelines about how to use the UCAPS^26^. Two training sessions were performed. The first training session consisted of an introduction and overview of the UCAPS. Videos were reviewed that exemplified behaviors for each item and observers discussed each video (https://animalpain.org/en/bois-dor-en/). The second training session was conducted in which observers assessed ten randomized videos of surgical castration pre and postoperative time-points of one Nelore and one Angus. These videos were excluded and were not part of the study. Each video was assessed, and scores were compared between the observers and discussed if results varied.
  For the real-time data collection, evaluators stood within 1 meter from the pen fence where animals were allocated to observe them. The pen had a group of three to four animals, identified with numbers, and assessed individually for each time-point. Evaluators did not talk to each other and minimized any movement during the assessment. After observing, the evaluators completed data collection in the following sequence: (a) ‘Would you provide rescue analgesia according to your clinical experience?’ If yes, mark ‘1’, if no, mark ‘0’ (this data was used for the rescue analgesia analysis); and (b) UCAPS scoring^26^. Real-time data collected occurred between March 18th and April 29th of 2019. Evaluators were aware of the time-points and procedure when scoring in real-time.
2. Video-recorded assessment
  Video was recorded using a high-definition video camera (Canon PowerShot SX50 HS, Oita, Japan) placed outside the outdoor pen, 1–2 m from the fence, using a camera tripod. A total of 95 videos (3 min in duration) were collected while animals were assessed using the real-time methodology. The video-recorded pain assessment was conducted more than six months after the real-time assessment by the same evaluators (A.R.O. and R.M.T.). Evaluators were masked to time-points, and order of observation was randomized. The evaluators watched the videos using separate computers and assessed the videos in the same randomized order. Upon watching the video, evaluators completed data collection in the same order as real-time assessment. Evaluators assessed videos for a maximum of 1 h a day to avoid fatigue. Video analysis occurred from November 22nd to December 22nd, 2019.

### Statistical analysis

Data was analyzed using R software within the integrated RStudio environment (Version 4.1.0; 2021-06-29; RStudio, Inc., Boston, MA, USA). The functions and packages used were presented in the format 'package::function' corresponding to the computer programming language in R. For all tests, a significance of 5% was considered. All figures were created with a color palette distinguishable by colorblind people (ggplot2::scale_colour_viridis_d). A minimum sample size of 11 subjects, with 0.80 of power and an alpha of 0.05 was calculated, based on Spearman correlation of rho = 0.764 between the UCAPS and Cow Pain Scale (http://biomath.info/power/ accessed on 10/01/2022).

Modeling was conducted to compare real-time versus video-recorded pain assessments for (i) UCAPS total, (ii) each UCAPS behavioral item, and (iii) rescue analgesia:

i. For UCAPS total, the histogram plot (stats::hist) (Fig. S1) and Cameron and Trivedi’s test (overdisp::overdisp) (Lambda t-test score = 4.687 and *p* < 0.00001) proved an overdispersion (excess of zeros), requiring a zero-inflated model^49^. Zero-inflated models combine Poisson and Bernoulli distributions in the fixed effects of the same model for a better fit of the data^37^. Therefore, a multilevel zero-inflated poisson model (glmmTMB::glmmTMB) was identified as the best fit compared with other types of models (Linear, Poisson and Negative Binomial models) according to the histogram (Fig. S1) and the Bayesian information criterion (stats::BIC), as proposed previously^34^. The UCAPS total was used as the response variable, while the fixed effects were composed of two components (Poisson and Bernoulli distributions), which is a special characteristic of the zero-inflated models^37^. Therefore, evaluators, breeds, and interaction between time-points and assessment methods were used as explanatory variables in the model fixed effects related to (Poisson distribution). Also, assessment methods were included as explanatory variable in the model fixed effects related to (Bernoulli distribution). The selection of fixed effects in the Poisson and Bernoulli component was guided by the Bayesian information criterion. Cattle was included as a crossed random effect of the model. The Bonferroni procedure was used to adjust the multiple comparisons to the post-hoc test (lsmeans::lsmeans and multcomp::cld).
ii. For each UCAPS behavioral items were used multilevel generalized model adjusted by Poisson distribution (lme4::glmer), based on data distribution and the Bayesian information criterion. Evaluators, breeds, and interaction between time-points and assessment methods were used as explanatory variables in the model fixed effects. Cattle was included as a crossed random effect of the model the Bonferroni was used to adjust the multiple comparisons to the post-hoc test.
iii. For rescue analgesia based on the clinical experience of the evaluators, a multilevel binomial logistic model (lme4::glmer) was used based on data distribution. Evaluators, breeds, and interaction between time-points and assessment methods were used as explanatory variables in the model fixed effects. Cattle was included as a crossed random effect of the model. The Bonferroni procedure was used to adjust the multiple comparisons to the post-hoc test.^51^

Bland–Altman test for repeated measures^50^ and Lin's concordance correlation coefficient (CCC)^51^ (SimplyAgree::agree_reps) were used to verify the agreement of UCAPS assessed in real-time and video-recorded methodology as proposed previously^37^. A simple linear regression (stats::lm) was conducted to analyze the proportion bias between both assessment methods^52^. Proportional bias represents an increase in the difference between the methods evaluated at higher or lower UCAPS total score^37,52^. Then, the difference of UCAPS total score between the two assessment methods was used as a response variable and the mean of UCAPS total score between the two methods was used as an explanatory variable. Heteroskedasticity was assessed by Breusch Pagan test (olsrr::ols_test_breusch_pagan)^53^.

Intraclass correlation coefficient (ICC), two-way random effects model, type agreement multiple evaluators/measurements, and its 95% confidence interval (CI) (irr::icc)^54,55^ was used to evaluate the inter-rater reliability of the UCAPS total score, considering the entire scale. The weighted kappa and its CI (biostatUZH::confIntKappa)^56^ was used to investigate the inter-rater reliability of the rescue analgesia.

Chi-square test (stats::chisq.test) was applied to analyze the relationship of the rescue analgesia between real-time and video-recorded assessment.

### Supplementary Information

Supplementary Information 1.

Supplementary Information 2.

Supplementary Information 3.

## Data Availability

All data analyzed during this study are included in this article in its supplementary information files.
