# Supplementary material for: Real-time and video-recorded pain assessment in beef cattle: clinical application and reliability in young, adult bulls undergoing surgical castration
Source: Sci Rep. 2024 Jul 2;14:15257. doi: 10.1038/s41598-024-65890-9 (PMC11220004; doi:10.1038/s41598-024-65890-9)
Supplement: Supplementary file 3 — Supplementary Information 3. [file 41598_2024_65890_MOESM3_ESM.docx]

# Supplementary material


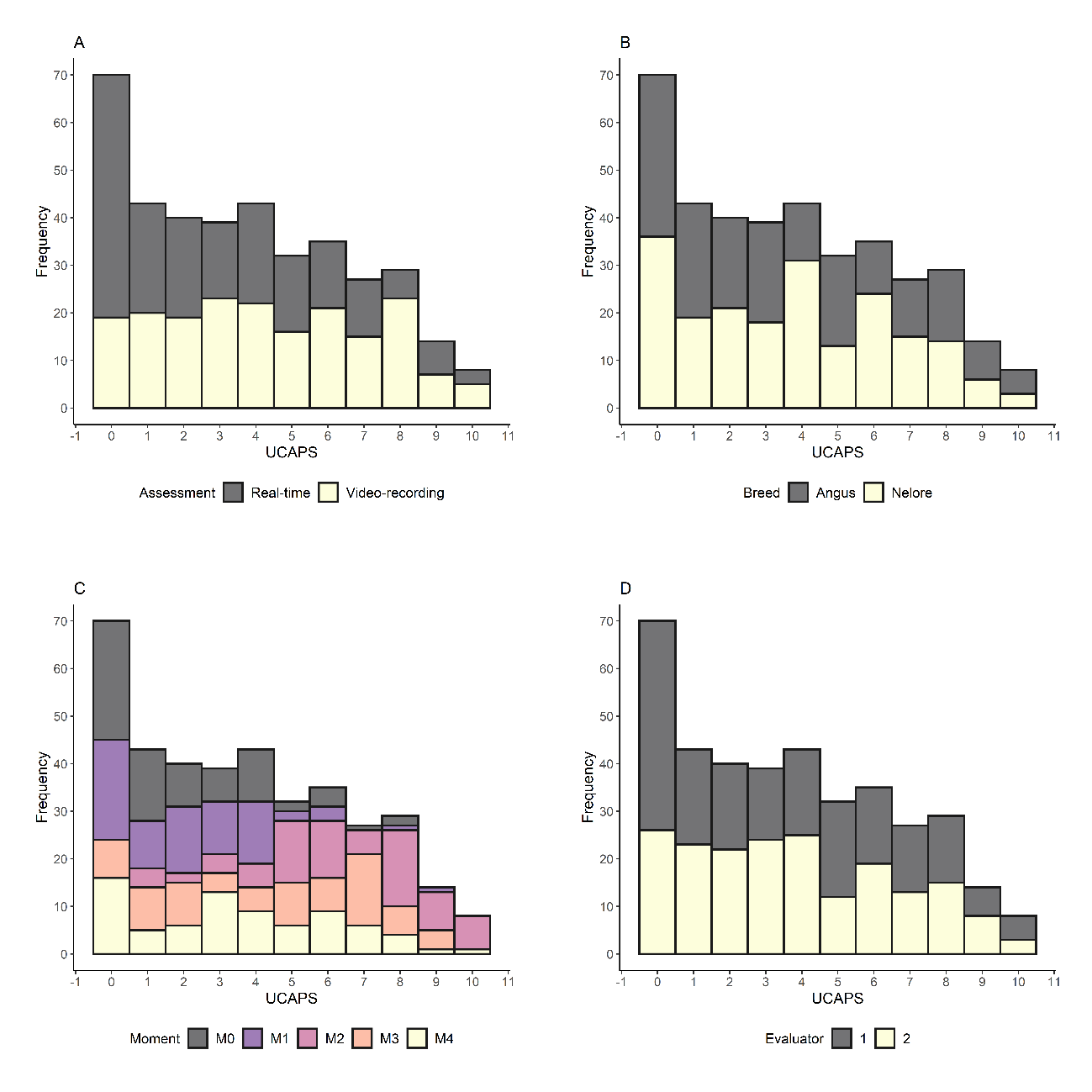


**Figure S1.** **Histogram of Unesp-Botucatu cattle composite acute pain scale (UCAPS) overdispersion per assessment method (A), breed (B), moment (C), and evaluator (D).**

**Table S1.** **Multilevel zero-inflated Poisson model findings using UCAPS as the response variable**. SE, standard-error; SD, standard-deviation.

| Fixed effects | Estimate | SE | Z-value | P-value |
| --- | --- | --- | --- | --- |
| Count component of the model | | | | |
| Linear coefficient (α) | 0.8197 | 0.1778 | 4.6111 | 4.01^-06^ |
|  |  |  |  |  |
| Slope coefficients (β) |  |  |  |  |
| AssessmentVideo-recorded | 0.1940 | 0.1954 | 0.9926 | 0.3209 |
| Time-pointM1 | -0.1933 | 0.2107 | -0.9174 | 0.3589 |
| Time-pointM2 | 1.0149 | 0.1694 | 5.9922 | 2.07^-09^ |
| Time-pointM3 | 0.5246 | 0.1810 | 2.8975 | 0.0038 |
| Time-pointM4 | 0.4563 | 0.1906 | 2.3946 | 0.0166 |
| BreedNelore | -0.0119 | 0.1074 | -0.1112 | 0.9114 |
| Evaluator2 | 0.0155 | 0.0557 | 0.2789 | 0.7803 |
| AssessmentVideo-recorded: Time-pointM1 | 0.3099 | 0.2599 | 1.1923 | 0.2331 |
| AssessmentVideo-recorded: Time-pointM2 | -0.2106 | 0.2156 | -0.9766 | 0.3288 |
| AssessmentVideo-recorded: Time-pointM3 | 0.1674 | 0.2264 | 0.7396 | 0.4595 |
| AssessmentVideo-recorded: Time-pointM4 | 0.0842 | 0.2367 | 0.3558 | 0.7220 |
|  |  |  |  |  |
| Logistic component of the model | | | | |
| Linear coefficient (α) | -1.3945 | 0.2301 | -6.0597 | 1.36^-09^ |
|  |  |  |  |  |
| Slope coefficients (β) |  |  |  |  |
| AssessmentVideo-recorded | -1.4269 | 0.4826 | -2.9566 | 0.0031 |
|  |  |  |  |  |
| Random effects | Estimate | SD | Number |  |
| Cattle | 0.0394 | 0.1985 | 19 |  |

**Table S2.** **Reliability of real-time and video recording assessment for Unesp-Botucatu cattle pain scale (UCAPS) items.** ICC, intraclass correlation coefficient; CI, 95% confidence interval. The interpretation of ICC or weighted kappa was ‘very good’ 0.81–1.0; ‘good’ 0.61–0.80; ‘moderate’ 0.41–0.60; ‘reasonable’ 0.21–0.4; and ‘poor’ <0.2 ^17^.

| **Variable** | **Method** | **Coefficient** | **Estimate** | **CI** | **P-value** |
| --- | --- | --- | --- | --- | --- |
| 1 Locomotion | Real life | Weighted Kappa | 0.73 | 0.57 – 0.82 | NA |
| 1 Locomotion | Video recording | Weighted Kappa | 0.52 | 0.33 – 0.67 | NA |
| 2 Interactive behaviour | Real life | Weighted Kappa | 0.56 | 0.37 – 0.71 | NA |
| 2 Interactive behaviour | Video recording | Weighted Kappa | 0.40 | 0.19 – 0.59 | NA |
| 3 Activity | Real life | Weighted Kappa | 0.43 | 0.26 – 0.59 | NA |
| 3 Activity | Video recording | Weighted Kappa | 0.50 | 0.32 – 0.66 | NA |
| 4 Appetite | Real life | Weighted Kappa | 0.89 | 0.79 – 0.94 | NA |
| 4 Appetite | Video recording | Weighted Kappa | 0.51 | 0.34 – 0.66 | NA |
| 5 Miscellaneous behaviour | Real life | Weighted Kappa | 0.57 | 0.38 – 0.73 | NA |
| 5 Miscellaneous behaviour | Video recording | Weighted Kappa | 0.60 | 0.45 – 0.73 | NA |
